# Crystal structure of bis­{μ_2_-2,2′-[(4,10-dimethyl-1,4,7,10-tetra­aza­cyclo­dodecane-1,7-di­yl)bis(meth­yl­ene)]bis­(4-oxo-4*H*-pyran-3-olato)}dicobalt­calcium bis­(perchlorate) 1.36-hydrate

**DOI:** 10.1107/S2056989017016693

**Published:** 2017-11-28

**Authors:** Patrizia Rossi, Eleonora Macedi, Paola Paoli, Luca Giorgi, Mauro Formica, Vieri Fusi

**Affiliations:** a Department of Industrial Engineering, University of Firenze, via Santa Marta 3, I-50139 Firenze, Italy; b Department of Pure and Applied Sciences, Lab of Supramolecular Chemistry, University of Urbino, via della Stazione, 4, I-61029 Urbino, Italy

**Keywords:** crystal structure, macrocycle, polyamine ligands, heterotrinuclear complexes, alkaline-earth cations, maltol

## Abstract

The title compound, [CaCo_2_(C_22_H_30_N_4_O_6_)_2_](ClO_4_)_2_·1.36H_2_O or {Ca[Co(H_–2_ **L1**)]_2_}·2ClO_4_·1.36H_2_O {where **L1** is 4,10-bis­[(3-hy­droxy-4-pyron-2-yl)meth­yl]-1,7-dimethyl-1,4,7,10-tetra­aza­cyclo­dodecane}, is a trinuclear complex whose asymmetric unit comprises a quarter of the {Ca[Co(H_–2_ **L1**)]_2_}^2+^ trinuclear complex, half of a perchlorate ion and 0.34-water mol­ecules. In the neutral [Co(H_–2_ **L1**)] moiety, the cobalt ion is hexa­coordinated in a trigonal–prismatic fashion by the surrounding N_4_O_2_ donor set. A Ca^2+^ cation holds together two neutral [Co(H_–2_ **L1**)] moieties and is octa­coordinated in a distorted trigonal–dodeca­hedral fashion by the surrounding O atoms belonging to the deprotonated oxide and carbonyl groups of two [Co(H_–2_ **L1**)] units. The coordination of the Co^II^ cation preorganizes **L1** and an electron-rich area forms, which is able to host hard metal ions. The comparison between the present structure and the previously published ones suggests a high versatility of this ligand; indeed, hard metal ions with different nature and dimensions lead to complexes having different stoichiometry (mono- and dinuclear monomers and trinuclear dimers) or even a polymeric structure. The heterotrinuclear Co^II^–Ca^II^–Co^II^ complexes are connected in three dimensions *via* weak C—H⋯O hydrogen bonds, which are also responsible for the inter­actions with the perchlorate anions and the lattice water mol­ecules. The perchlorate anion is disordered about a twofold rotation axis and was refined giving the two positions a fixed occupancy factor of 0.5. The crystal studied was refined as a two-component inversion twin [BASF parameter = 0.14 (4)].

## Abstract 2

The title compound is a new heterotrinuclear Co^II^–Ca^II^–Co^II^ dimer of **L1. L1** undergoes a cobalt-driven preorganization, leading to the formation of an electron-rich area able to host a hard metal ion such as Ca^II^. In the dimer, two neutral [Co(H_–2_ **L1**)] moieties, held together by the Ca^II^ ion, are rotated by 90°. The trinuclear complexes form layers perpendicular to the *c* axis; the perchlorate anions are located between the layers and inter­act with the complexes, as well as the lattice water mol­ecules.

## Chemical context   

Polynuclear metal complexes have long been studied due to their versatility. They find applications in many fields, ranging from mol­ecular recognition to transport and catalysis (Gokel & Barbour, 2017 ▸; Weber & Gokel, 2012 ▸; Ambrosi *et al.*, 2007*a* ▸,*b* ▸, 2008 ▸, 2009*a* ▸,*b* ▸; Martell & Hancock, 1996 ▸; Voegtle, 1996 ▸; Zelewsky, 1996 ▸; Lehn, 1988 ▸), to name just a few. Moreover, they find applications in the field of bioinorganic chemistry (Fanelli *et al.*, 2016 ▸; Marchetti *et al.*, 2015 ▸; Patra *et al.*, 2014 ▸), for instance as anti­cancer agents (Bruijnincx & Sadler, 2008 ▸) and artificial metalloproteases (Suh & Chei, 2008 ▸).

On the other hand, hard metal ions also find applications in the biological field. Both rare earth and alkaline earth metal ions are used in the biomedical field, in bioassays and bio­imaging applications (Xiao *et al.*, 2016 ▸; Yin *et al.*, 2015 ▸; DaCosta *et al.*, 2014 ▸; Merbach *et al.*, 2013 ▸; Di Bernardo *et al.*, 2012 ▸; Price *et al.*, 2012 ▸). Furthermore, hard metal ions are quite difficult to bind in water because they need a high coordination number without usually showing specific coordination requirements, issues that could be overcome using preorg­anized receptors bearing oxygenated donor sites. It follows that systems able to bind hard metal ions, both in aqueous solution and in the solid state, are very attractive. Indeed, they have found applications in fields ranging from new materials to medicinal chemistry (Blindauer *et al.*, 2017 ▸; Esteves *et al.*, 2016 ▸; Lomidze *et al.*, 2016 ▸; Yang *et al.*, 2014 ▸; Price *et al.*, 2012 ▸; Pasatoiu *et al.*, 2011 ▸; Pasatoiu *et al.*, 2010 ▸; Aime *et al.*, 2006 ▸; Bernot *et al.*, 2006 ▸; Gatteschi *et al.*, 2006 ▸; Malandrino & Fragalà, 2006 ▸; Terai *et al.*, 2006 ▸).

Ligand **L1** {4,10-bis­[(3-hy­droxy-4-pyron-2-yl)meth­yl]-1,7-dimethyl-1,4,7,10-tetra­aza­cyclo­dodeca­ne} is a Maltol-based macrocycle (Amatori *et al.*, 2012 ▸) capable of forming a mononuclear Co^II^ species where both side-arms are forced by the transition metal ion to move and locate on the same part with respect to the macrocyclic plane (Borgogelli *et al.*, 2013 ▸). Such a cobalt-driven preorganization allows the formation of an electron-rich area formed by the four converging oxygen atoms of the two maltolate functions of **L1**, suitable to host hard metal ions such as *Ln* ^III^ (*Ln* = Gd, Eu; Benelli *et al.*, 2013 ▸; Rossi *et al.*, 2017 ▸), Na^I^ (Borgogelli *et al.*, 2013 ▸) and Ba^II^ (Paoli *et al.*, 2017 ▸). The resulting heteropolynuclear systems differ in the number of the complexes involved in the coordination, depending on the nature of the hard cation. Indeed, the coordination of the hard ion leads to Co^II^–*Ln* ^III^–Co^II^ heterotrinuclear dimers, a Na^I^–Co^II^ heterodinuclear monomer and a Ba^II^–Co^II^ heterodinuclear metal coordination polymer.
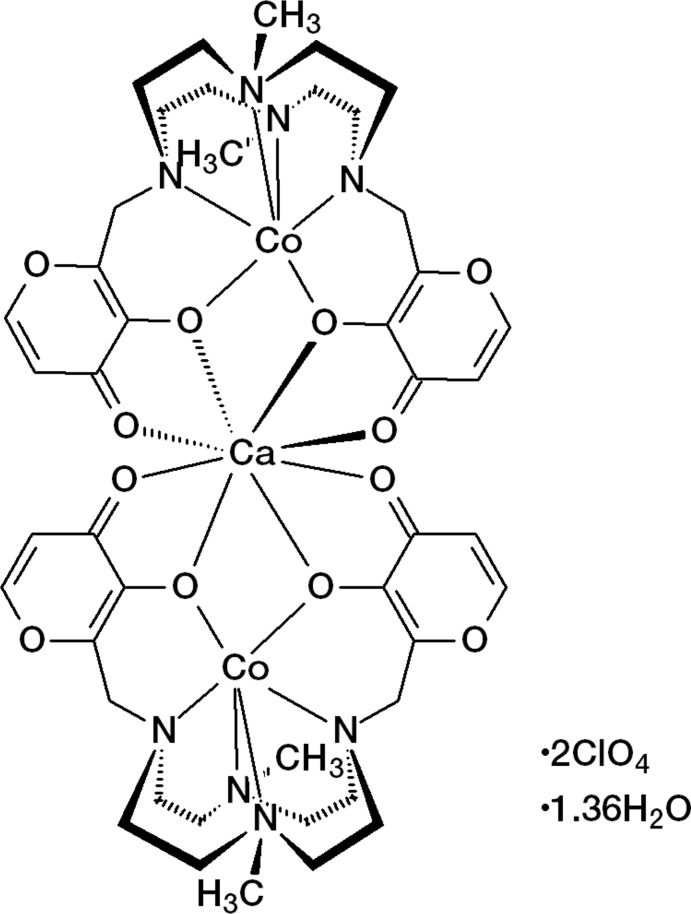


Herein we present a Co^II^–Ca^II^–Co^II^ heterotrinuclear dimer of **L1** and a brief comparison with the previous **L1**-containing structures, highlighting the high versatility of this ligand.

## Structural commentary   

**Figure 1:**
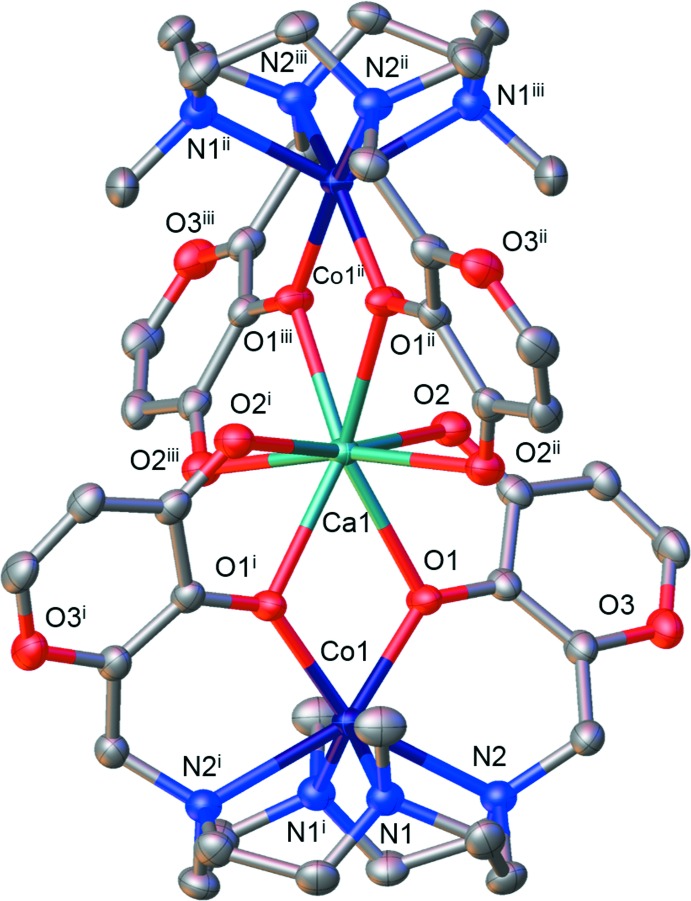
The mol­ecular structure of the {Ca[Co(H_–2_ **L1**)]_2_}^2+^ cation, with the atom labelling and 30% probability displacement ellipsoids. H atoms have been omitted for clarity. [Symmetry codes: (i) −*x*, −*y*, *z*; (ii) *y*, −*x*, −*z*; (iii) −*y*, *x*, −*z*.]

The title compound is a trinuclear complex cation of formula {Ca[Co(H_–2_ **L1**)]_2_}·2ClO_4_·1.36H_2_O and crystallizes in the tetra­gonal system in space group *I* 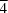. In the {Ca[Co(H_-2_ **L1**)]_2_}^2+^ trinuclear complex (Fig. 1 ▸), two neutral [Co(H_-2_ **L1**)] moieties are held together by the Ca^2+^ cation, which is coordinated by oxygen atoms provided by the maltolate groups of the two complexes. The asymmetric unit comprises a quarter of the {Ca[Co(H_–2_ **L1**)]_2_}^2+^ trinuclear complex, half of a perchlorate ion and 0.34 water mol­ecules. The two halves of each cobalt complex are related by a twofold rotation axis, the cobalt ion lying on the symmetry element. The two cobalt complexes are then related by a fourfold rotoinversion axis, the calcium ion lying on the symmetry element. The disordered perchlorate ion and the water mol­ecule lie on a twofold axis, with the chlorine atom (for ClO_4_^−^) and the oxygen atom (for H_2_O) lying on the symmetry element.

**Figure 2:**
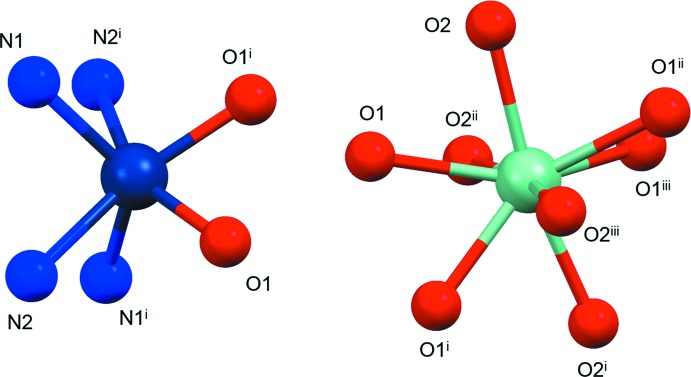
Coordination polyhedra around the cobalt (left) and calcium (right) ions. [Symmetry codes: (i) −*x*, −*y*, *z*; (ii) *y*, −*x*, −*z*; (iii) −*y*, *x*, −*z*.]

In the neutral [Co(H_–2_ **L1**)] moiety, the Co^2+^ ion is hexa­coordinated by four nitro­gen atoms of the macrocyclic base and two deprotonated hydroxyl oxygen atoms provided by both the maltolate rings of the ligand; it exhibits a distorted trigonal–prismatic geometry (Muetterties & Guggenberger, 1974 ▸), with the N1,N2^i^,O1^i^/N1^i^,N2,O1 atoms [symmetry code: (i) −*x*, −*y*, *z*] defining the two triangular faces, which are parallel within 15.6 (2)° (Fig. 2 ▸, left). The cobalt ion is displaced 1.064 (1) Å above the mean plane defined by the four nitro­gen atoms of the tetra­aza­macrocycle [maximum deviation of 0.044 (6) Å for N1]; according to the Cambridge Structural Database (CSD, Version 5.38, May 2017; Groom *et al.*, 2016 ▸) such distance falls, together with the Co—N(CH_3_) and Co—O bond distances (Table 1 ▸), in the expected range for Co-[12]aneN_4_ complexes where the cobalt ion is hexa­coordinated with an N_4_O_2_ donor set. The Co—N(Maltol) bond distances, by contrast, are beyond this range (Table 1 ▸) but are in line with those reported for other Co—**L1** complexes [Co—N(Maltol): range 2.26–2.44 Å; Co—N(CH_3_) range: 2.13—2.22 Å; Benelli *et al.*, 2013 ▸; Borgogelli *et al.*, 2013 ▸; Rossi *et al.*, 2017 ▸; Paoli *et al.*, 2017 ▸].

The conformation of the [12]aneN_4_ macrocycle is the usual [3333]C-corners one (Meurant, 1987 ▸) with the *trans* nitro­gen distances in agreement with those reported in the CSD for this conformation type, but with the N2⋯N2^i^ distance being longer than N1⋯N1^i^ by 0.32 Å [Table 1 ▸, symmetry code: (i) −*x*, −*y*, *z*], as found only in 12% of cases (88%: Δ < 0.32 Å; 12%: Δ > 0.32 Å). This is probably due to the fact that the Maltol units linked to the nitro­gen atoms are involved in chelate six-membered rings, which stiffen the system and force those nitro­gen atoms to move farther apart.

The mean planes of the two maltolate rings of the neutral [Co(H_–2_ **L1**)] moiety form a dihedral angle of about 55°, while the dihedral angle between the N1,N2,N1^i^,N2^i^ [symmetry code: (i) −*x*, −*y*, *z*] and maltolate ring mean planes is about 63°. The distance between the maltolate ring centroids is 7.8463 (3) Å. The dimension of the binding area defined by the four oxygen donor atoms of the ligand is roughly estimated by the distance separating the opposite O1⋯O2^i^ [symmetry code: (i) −*x*, −*y*, *z*] atoms (and the other symmetry-related oxygen atoms), which is 4.315 (6) Å. Notably, such a distance is longer than those retrieved for analogous trinuclear complexes (opposite O⋯O distances range: 3.98–4.22 Å; Benelli *et al.*, 2013 ▸; Rossi *et al.*, 2017 ▸), while it is shorter than those retrieved for the one-dimensional coordination polymer of **L1** (opposite O⋯O distances: 4.5 Å; Paoli *et al.*, 2017 ▸) and the mononuclear complex of **L1** (opposite O⋯O distances: 4.49 Å; Borgogelli *et al.*, 2013 ▸). As for the dinuclear complex of **L1** (Borgogelli *et al.*, 2013 ▸), the opposite O⋯O distances of the binding area are quite different from each other (4.12 and 4.42 Å), and are, respectively, shorter and longer than the corresponding distance in the title compound.

The coordination polyhedron around the Ca^2+^ ion can be described as a distorted trigonal dodeca­hedron (Muetterties & Guggenberger, 1974 ▸), with all eight deprotonated hydroxyl and carbonyl oxygen atoms of the two [Co(H_–2_ **L1**)] moieties of the trinuclear complex situated at the corners of the polyhedron (Fig. 2 ▸, right). The maltolate unit acts as a bidentate ligand through the hydroxyl oxygen atom, which bridges the Ca^II^ and Co^II^ cations. All the Ca—O distances are in agreement with data found in the CSD.

**Figure 3:**
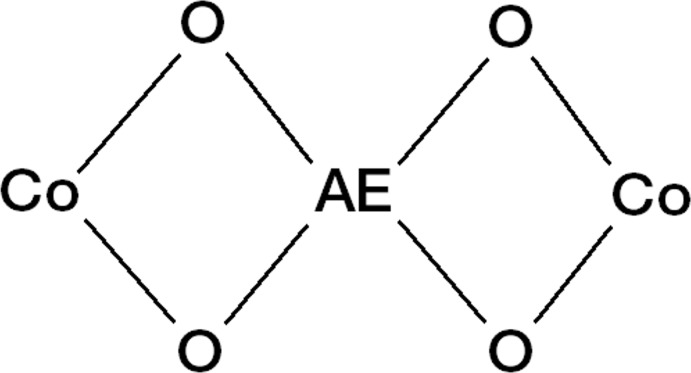
Fragment searched in the CSD. [AE = alkaline-earth metal ion.]

The Co^2+^ and Ca^2+^ cations are located 3.727 (1) Å apart from each other and, because of the symmetry of the system, the line connecting the three cations (Co^II^–Ca^II^–Co^II^) is normal to the mean plane described by the four nitro­gen atoms of the macrocycle (Fig. 1 ▸). The values for the Co⋯Ca distance and the Co—O1—Ca angle are in agreement with data ranges found in the CSD, even if they fall in non-populated regions (only ten hits – corresponding to twenty distances or angle values – are retrieved when the Co–O–Ca fragment is searched). The Co⋯Co^ii^ distance and the Co–Ca–Co^ii^ angle value [symmetry code: (ii) *y*, −*x*, −*z*] can only be compared with the single hit containing a cobalt-μ_2_-oxygen-calcium-μ_2_-oxygen-cobalt motif (Fig. 3 ▸) deposited in the CSD (refcode: DAPNOA; Li *et al.*, 2017 ▸), which shows a shorter Co⋯Co distance (6.25 Å) and a smaller Co⋯Ca⋯Co angle value (132°) with respect to the title compound. When all alkaline-earth ions instead of calcium are considered in the fragment searched in the CSD (Fig. 3 ▸), both the Co⋯Co distance and Co⋯Ca⋯Co angle values fall within the expected range.

As a result of the symmetry of the system, the two [Co(H_–2_ **L1**)] complexes in the {Ca[Co(H_–2_ **L1**)]_2_}^2+^ cation are rotated by 90°, as indicated by the angle between the two mean planes defined by the Co1,O1,O1^i^,Ca1 and Co^ii^,O1^ii^,O1^iii^,Ca1 atoms [symmetry codes: (i) −*x*, −*y*, *z*; (ii) *y*, −*x*, −*z*; (iii) −*y*, *x*, −*z*; Fig. 1 ▸]. Such an angle value falls in the most populated region for the cobalt-μ_2_-oxygen-AE-μ_2_-oxygen-cobalt fragment (AE = alkaline-earth ion).

Finally, the shortest Co⋯Co/Co⋯Ca/Ca⋯Ca distances between metal cations belonging to different {Ca[Co(H_–2_ **L1**)]_2_}^2+^ units are 8.9799 (4)/9.7227 (5)/8.9799 (4) Å.

**Figure 4:**
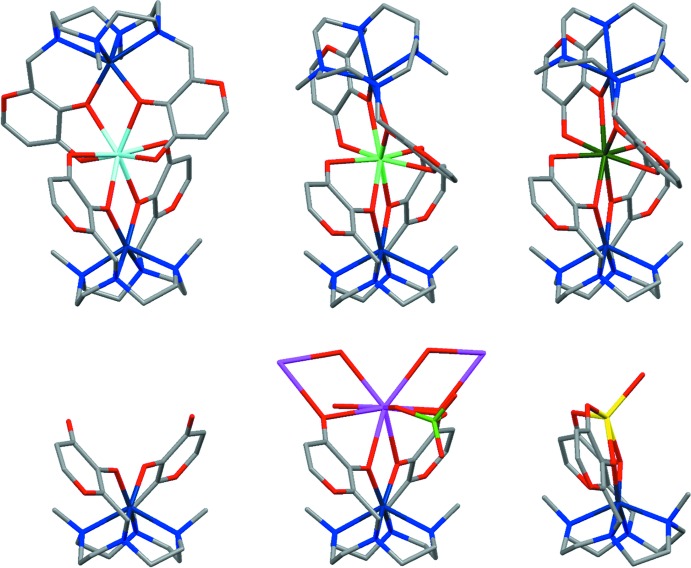
Comparison between the overall shapes of the present structure and the other Co-containing structures of **L1**. Top line, from left to right: Co^II^–Ca^II^–Co^II^, Co^II^–Eu^III^–Co^II^ (Rossi *et al.*, 2017 ▸), Co^II^–Gd^III^–Co^II^ (refcode: FEZBUJ) complexes; bottom line, from left to right: Co^II^ species (refcode: WELGEB), Ba^II^–Co^II^ coordination polymer (refcode: ZELBAW), Na^I^–Co^II^ complex (refcode: WELGOL).

In the present structure and in all the Co-containing structures of **L1** published up to now, the cobalt complexes are well superimposable with each other, but for that belonging to the Na^I^–Co^II^ heterodinuclear complex (r.m.s. deviation values of 0.788 Å and within 0.301 Å for the superimposition of the title compound with the Na^I^–Co^II^ complex and with all other structures, respectively), where the two maltolate rings show a different arrangement, both rings being tilted toward the same direction (instead of opposite directions) with respect to the cobalt-μ_2_-oxygen-hard metal mean plane (*M* = Na^I^, Ca^II^, Ba^II^, Gd^III^, Eu^III^; in the case of the mononuclear Co^II^ species, with respect to the cobalt–μ_2_-oxygen mean plane; Fig. 4 ▸). Moreover, when considering the heterotrinuclear complexes only, the superimposition of the Co^II^–Ca^II^–Co^II^ dimer with the whole structures of the Co^II^–*Ln* ^III^–Co^II^ dimers (*Ln* ^III^ = Gd^III^, Eu^III^) shows high r.m.s. deviation values (1.7 Å), in agreement with a different mutual disposition of the two subunits in the dimers.

The electron-rich area, which forms following the cobalt-driven preorganization of **L1**, is able to host hard metal ions with different dimensions and coordination requirements, leading to complexes having different stoichiometry (mono- and dinuclear monomers and trinuclear dimers) or even a polymeric structure (Fig. 4 ▸). In the case of the Na^I^–Co^II^ structure, a monomer forms, probably because of the lower ionic charge and coordination number (CN) of the Na^I^ cation (CN: 5, Na^+^ ionic radius: 1.00 Å; Shannon, 1976 ▸) with respect to the other cations. Indeed, the low ionic charge and coord­ination number allow the stabilization of the ion with only one [Co(H_–2_ **L1**)] moiety. In the case of the Ba^II^–Co^II^ structure, the Ba^II^ cation shows the highest coordination number (CN: 9, Ba^2+^ ionic radius: 1.47 Å; Shannon, 1976 ▸) in the series of structures, and the cationic fragment shows the largest binding area, which is necessary to accommodate such large ionic dimensions. In the case of the heterotrinuclear structures, all of the Gd^III^, Eu^III^ and Ca^II^ cations have the same coordination number (CN: 8) and similar ionic radii (1.053, 1.066 and 1.12 Å for Gd^III^, Eu^III^ and Ca^II^, respectively; Shannon, 1976 ▸): two [Co(H_–2_ **L1**)] units are needed to stabilize the high ionic charge and fully satisfy the coordination requirements of the cations.

## Supra­molecular features   

In the crystal, the heterotrinuclear Co^II^–Ca^II^–Co^II^ complexes are connected in the three dimensions *via* weak C—H⋯O hydrogen bonds (Desiraju & Steiner, 1999 ▸).

**Figure 5:**
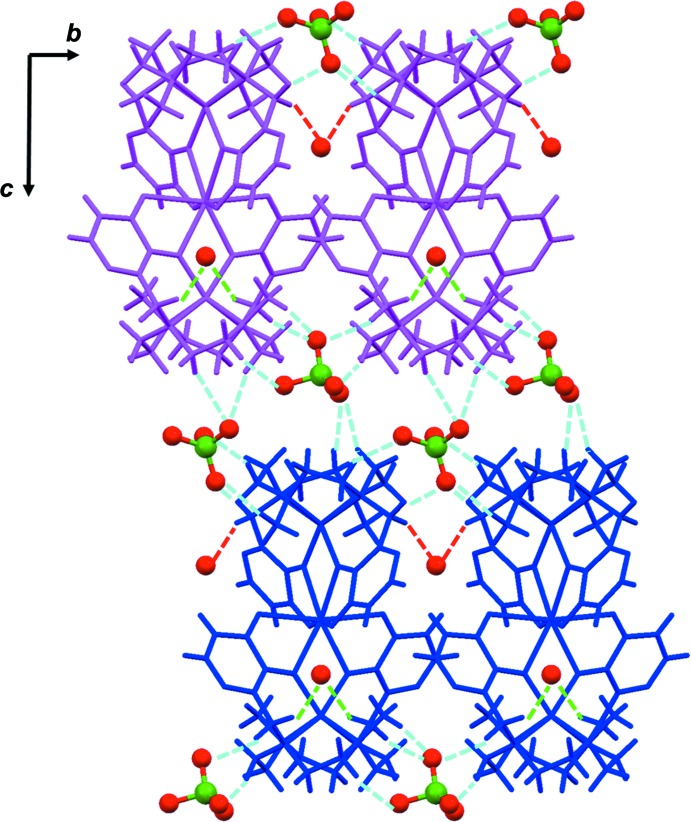
Crystal packing of the title compound viewed along the *a* axis. Staggered layers of complexes (in magenta and blue) perpendicular to the *c* axis are present, which are inter­connected thanks to hydrogen bonds in the *c-*axis direction. The perchlorate anions are located between the layers. Inter­actions with water mol­ecules are also shown. Hydrogen bonds involving ClO_4_^−^ anions are depicted as light-blue dotted lines. Hydrogen bonds involving water mol­ecules are depicted as green (along the *a* axis) and red (along the *b* axis) dotted lines. The ClO_4_^−^ anions and water mol­ecules are depicted in ball-and-stick mode.

**Figure 6:**
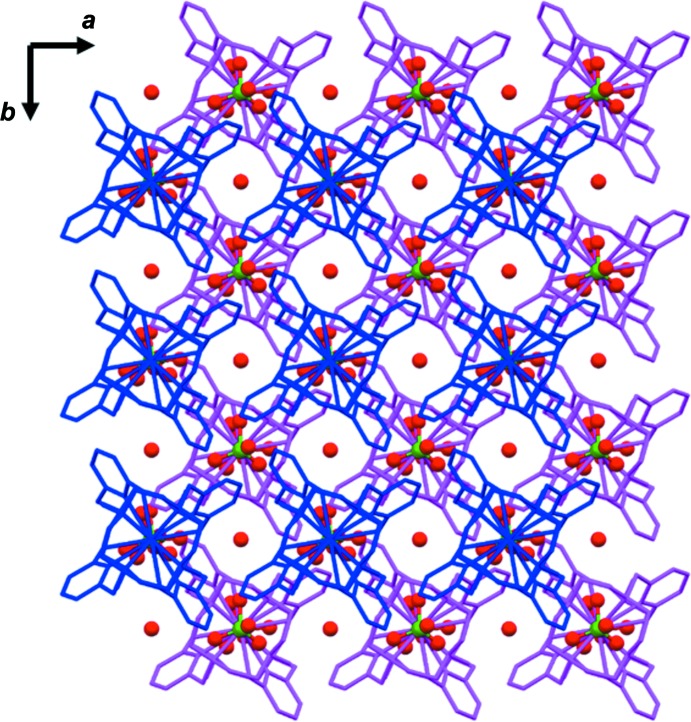
Crystal packing of the title compound viewed along the *c* axis. Staggered layers of complexes (in magenta and blue) perpendicular to the *c* axis are visible. The ClO_4_^−^ anions and water mol­ecules are depicted in ball-and-stick mode.

The perchlorate anion inter­acts with five complexes: four out of five (magenta in Figs. 5 ▸ and 6 ▸) are connected to form a layer perpendicular to the *c* axis, the fifth complex also belongs to a layer (blue in Figs. 5 ▸ and 6 ▸) perpendicular to the *c* axis, adjacent layers being staggered relative to one other (Fig. 6 ▸). All inter­actions are weak C—H⋯O—Cl hydrogen bonds (Table 2 ▸) involving the methyl­ene hydrogen atoms of the macrocycle. The perchlorate anions are located between the layers (Fig. 5 ▸).

Water mol­ecules also inter­act with the complexes *via* weak C—H⋯O hydrogen bonds (Table 2 ▸) along the *a* and *b* axes (Fig. 5 ▸). These inter­actions also involve the methyl­ene hydrogen atoms of the macrocycle.

## Synthesis and crystallization   

Compound **L1** was obtained following the previously reported synthetic procedure (Amatori *et al.*, 2012 ▸).

To obtain the title compound, {Ca[Co(H_–2_ **L1**)]_2_}·2ClO_4_·1.36H_2_O, 0.1 mmol of CoCl_2_· 6H_2_O in water (10 ml) were added to an aqueous solution (20 ml) containing 0.1 mmol of **L1**·3HClO_4_·H_2_O. The solution was adjusted to pH 7 with 0.1 *M* N(CH_3_)_4_OH and then 0.05 mmol of CaCl_2_ were added. The solution was saturated with NaClO_4_. The title compound quickly precipitated as a microcrystalline pink solid. Crystals suitable for X-ray analysis were obtained by slow evaporation of a more diluted aqueous solution.

## Refinement   

Crystal data, data collection and structure refinement details are summarized in Table 3 ▸.

All hydrogen atoms of the macrocycle were positioned geometrically and refined as riding with C—H = 0.95–0.99 Å with *U*_iso_(H) = 1.5*U*_eq_(C-meth­yl) and = 1.2*U*_eq_(C) for other H atoms.

The perchlorate anion is disordered about a twofold rotation axis and was refined giving the two positions a fixed occupancy factor of 0.5. The chlorine atom is located on a twofold rotation axis.

The oxygen atom of the water mol­ecule lies on a twofold rotation axis, the refined occupancy factor is 0.34 (2); the hydrogen atoms were not found in the difference-Fourier map and they were not introduced in the refinement.

All non-hydrogen atoms were refined anisotropically: as for the disordered perchlorate anion, the SIMU instruction was used to restrain the anisotropic displacement parameters of the disordered atoms, while the ISOR instruction was used to restrain the anisotropic displacement parameters of the isolated water oxygen atom.

The structure was refined as a two-component inversion twin [BASF parameter = 0.14 (4)].

## Supplementary Material

Crystal structure: contains datablock(s) global, I. DOI: 10.1107/S2056989017016693/xi2003sup1.cif

Structure factors: contains datablock(s) I. DOI: 10.1107/S2056989017016693/xi2003Isup2.hkl

Supporting information file. DOI: 10.1107/S2056989017016693/xi2003Isup3.mol

CCDC reference: 1586509

Additional supporting information:  crystallographic information; 3D view; checkCIF report

## Figures and Tables

**Table 1:** Selected bond lengths and angles (Å, °)

Co1—N1	2.192 (7)
Co1—N2	2.375 (7)
Co1—O1	2.060 (4)
Ca1—O1	2.429 (4)
Ca1—O2	2.469 (4)
N1⋯N1^i^	3.881 (9)
N2⋯N2^i^	4.206 (10)
Co1⋯Ca1	3.727 (1)
Co1⋯Co1^ii^	7.454 (2)
	
N1—Co1—N1^i^	124.5 (2)
N1—Co1—N2	78.0 (2)
N1—Co1—N2^i^	77.0 (3)
N2—Co1—N2^i^	124.6 (3)
O1—Co1—N1	121.2 (2)
O1—Co1—N1^i^	102.9 (2)
O1—Co1—N2	81.6 (2)
O1—Co1—N2^i^	152.4 (2)
O1—Co1—O1^i^	74.3 (2)
O1—Ca1—O1^i^	61.7 (2)
O1—Ca1—O1^ii^	137.5 (1)
O1—Ca1—O2	67.1 (1)
O1—Ca1—O2^i^	123.5 (1)
O1—Ca1—O2^ii^	73.9 (1)
O1—Ca1—O2^iii^	96.6 (1)
O2—Ca1—O2^i^	169.1 (2)
Co1—O1—Ca1	112.0 (2)

Symmetry codes: (i) −*x*, −*y*, *z*; (ii) *y*, −*x*, −*z*; (iii) −*y*, *x*, −*z*.

**Table 2:** Hydrogen-bond geometry (Å, °) Note that both models of the disordered perchlorate anion form the same inter­actions; only one value for each inter­action involving oxygen atoms of the ClO_4_^−^ anion is therefore reported.

*D*—H⋯*A*	*D*—H	H⋯*A*	*D*⋯*A*	*D*—H⋯*A*
C1^iv^—H1*A* ^iv^⋯O3*C*	0.99	2.64	3.56 (2)	156
C2^iii^—H2*A* ^iii^⋯O4*C*	0.99	2.68	3.55 (2)	147
C4^iv^—H4*C* ^iv^⋯O1*C*	0.98	2.50	3.47 (2)	167
C5^v^—H5*A* ^v^⋯O2*C*	0.99	2.59	3.48 (2)	148
C5^iii^—H5*B* ^iii^⋯O4*C*	0.99	2.35	3.29 (2)	157
C6—H6*A*⋯O1*W*	0.99	2.35	3.23 (9)	149
C6^vi^—H6*B* ^vi^⋯O1*C*	0.99	2.44	3.37 (2)	158
C6^vii^—H6*B* ^vii^⋯O1*C*	0.99	2.55	3.51 (2)	164

Symmetry codes: (iii) −*y*, *x*, −*z*; (iv) −*x* + 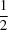, −*y* + 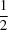, *z* + 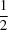; (v) *x* − 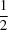, *y* − 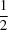, *z* + 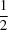; (vi) −*x* − 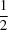, −*y* + 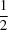, *z* + 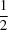; (vii) *x* + 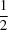, *y* − 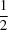, *z* + 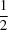.

**Table 3:** Experimental details

Crystal data	
Chemical formula	[CaCo_2_(C_22_H_30_N_4_O_6_)_2_](ClO_4_)_2_·1.36H_2_O
*M*_r_	1271.92
Crystal system, space group	Tetragonal, *I* 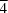
Temperature (K)	100
*a*, *c* (Å)	8.9799 (4), 32.555 (3)
*V* (Å^3^)	2625.2 (3)
*Z*	2
Radiation type	Mo *K*α
μ (mm^−1^)	0.92
Crystal size (mm)	0.46 × 0.38 × 0.18

Data collection	
Diffractometer	Oxford Diffraction Xcalibur Sapphire3
Absorption correction	Multi-scan (*CrysAlis RED*; Oxford Diffraction, 2008 ▸)
*T*_min_, *T*_max_	0.557, 1.000
No. of measured, independent and observed [*I* > 2σ(*I*)] reflections	3291, 2340, 1386
*R*_int_	0.034
(sin θ/λ)_max_ (Å^−1^)	0.681

Refinement	
*R*[*F* ^2^ > 2σ(*F* ^2^)], *wR*(*F* ^2^), *S*	0.043, 0.081, 0.86
No. of reflections	2340
No. of parameters	200
No. of restraints	30
H-atom treatment	H-atom parameters constrained
Δρ_max_, Δρ_min_ (e Å^−3^)	0.35, −0.25
Absolute structure	Refined as an inversion twin
Absolute structure parameter	0.14 (4)

Computer programs: *CrysAlis CCD* (Oxford Diffraction, 2008 ▸), *CrysAlis RED* (Oxford Diffraction, 2008 ▸), *SIR2014* (Burla *et al.*, 2015 ▸) and *SHELXL2014* (Sheldrick, 2015 ▸).

## supplementary crystallographic information

### Crystal data

**Table:**

[CaCo_2_(C_22_H_30_N_4_O_6_)_2_](ClO_4_)_2_·1.36H_2_O	*D*_x_ = 1.609 Mg m^−^^3^
*M**_r_* = 1271.92	Mo *K*α radiation, λ = 0.71073 Å
Tetragonal, *I*4	Cell parameters from 1087 reflections
*a* = 8.9799 (4) Å	θ = 3.7–28.8°
*c* = 32.555 (3) Å	µ = 0.92 mm^−^^1^
*V* = 2625.2 (3) Å^3^	*T* = 100 K
*Z* = 2	Prism, pink
*F*(000) = 1318	0.46 × 0.38 × 0.18 mm

### Data collection

**Table:**

Oxford Diffraction Xcalibur Sapphire3 diffractometer	2340 independent reflections
Radiation source: fine-focus sealed X-ray tube, Enhance (Mo) X-ray Source	1386 reflections with *I* > 2σ(*I*)
Graphite monochromator	*R*_int_ = 0.034
Detector resolution: 16.4547 pixels mm^-1^	θ_max_ = 29.0°, θ_min_ = 3.8°
ω scans	*h* = −12→10
Absorption correction: multi-scan (CrysAlis RED; Oxford Diffraction, 2008)	*k* = −12→9
*T*_min_ = 0.557, *T*_max_ = 1.000	*l* = −43→30
3291 measured reflections	

### Refinement

**Table:**

Refinement on *F*^2^	Hydrogen site location: inferred from neighbouring sites
Least-squares matrix: full	H-atom parameters constrained
*R*[*F*^2^ > 2σ(*F*^2^)] = 0.043	*w* = 1/[σ^2^(*F*_o_^2^) + (0.0355*P*)^2^] where *P* = (*F*_o_^2^ + 2*F*_c_^2^)/3
*wR*(*F*^2^) = 0.081	(Δ/σ)_max_ < 0.001
*S* = 0.86	Δρ_max_ = 0.35 e Å^−^^3^
2340 reflections	Δρ_min_ = −0.25 e Å^−^^3^
200 parameters	Absolute structure: Refined as an inversion twin
30 restraints	Absolute structure parameter: 0.14 (4)

### Special details

**Table:**

Geometry. All esds (except the esd in the dihedral angle between two l.s. planes) are estimated using the full covariance matrix. The cell esds are taken into account individually in the estimation of esds in distances, angles and torsion angles; correlations between esds in cell parameters are only used when they are defined by crystal symmetry. An approximate (isotropic) treatment of cell esds is used for estimating esds involving l.s. planes.
Refinement. Refined as a 2-component inversion twin.

### Fractional atomic coordinates and isotropic or equivalent isotropic displacement parameters (Å^2^)

**Table:**

	*x*	*y*	*z*	*U*_iso_*/*U*_eq_	Occ. (<1)
Co1	0.0000	0.0000	−0.11449 (3)	0.0355 (3)	
Ca1	0.0000	0.0000	0.0000	0.0350 (5)	
O1	−0.1168 (4)	0.0747 (5)	−0.06406 (11)	0.0431 (10)	
N1	0.1622 (8)	0.1428 (7)	−0.1458 (2)	0.0439 (16)	
C1	0.0890 (10)	0.2753 (10)	−0.1643 (3)	0.062 (3)	
H1A	0.1533	0.3164	−0.1862	0.075*	
H1B	0.0761	0.3530	−0.1430	0.075*	
O2	−0.2696 (5)	0.0475 (5)	0.00717 (15)	0.0510 (13)	
N2	−0.1567 (7)	0.1741 (8)	−0.14838 (19)	0.0464 (16)	
C2	−0.0591 (10)	0.2353 (12)	−0.1819 (2)	0.055 (2)	
H2A	−0.0466	0.1599	−0.2038	0.066*	
H2B	−0.1062	0.3247	−0.1941	0.066*	
O3	−0.4188 (5)	0.3327 (5)	−0.07952 (15)	0.0562 (12)	
C3	−0.2837 (8)	0.0927 (9)	−0.1656 (3)	0.048 (2)	
H3A	−0.3229	0.1474	−0.1897	0.058*	
H3B	−0.3639	0.0865	−0.1448	0.058*	
C4	0.2708 (9)	0.1931 (10)	−0.1142 (3)	0.066 (2)	
H4A	0.2181	0.2471	−0.0924	0.099*	
H4B	0.3213	0.1063	−0.1024	0.099*	
H4C	0.3446	0.2589	−0.1269	0.099*	
C5	0.2389 (11)	0.0621 (10)	−0.1785 (3)	0.055 (3)	
H5A	0.3289	0.1183	−0.1866	0.066*	
H5B	0.1727	0.0555	−0.2027	0.066*	
C6	−0.2118 (8)	0.3006 (8)	−0.1240 (2)	0.047 (2)	
H6A	−0.1266	0.3648	−0.1163	0.056*	
H6B	−0.2804	0.3607	−0.1411	0.056*	
C7	−0.2900 (7)	0.2535 (7)	−0.0865 (2)	0.0452 (16)	
C8	−0.2406 (6)	0.1491 (6)	−0.0589 (2)	0.0378 (14)	
C9	−0.3217 (7)	0.1295 (7)	−0.0201 (2)	0.0445 (17)	
C10	−0.4591 (7)	0.2091 (7)	−0.0171 (2)	0.0491 (18)	
H10	−0.5227	0.1926	0.0057	0.059*	
C11	−0.4987 (8)	0.3054 (9)	−0.0458 (2)	0.061 (2)	
H11	−0.5894	0.3582	−0.0422	0.073*	
Cl1	0.0000	0.0000	0.29295 (6)	0.0614 (6)	
O1C	0.018 (3)	0.0426 (14)	0.3345 (2)	0.069 (3)	0.5
O2C	−0.0574 (17)	−0.1450 (14)	0.2799 (4)	0.090 (3)	0.5
O3C	0.1595 (14)	−0.0078 (17)	0.2807 (4)	0.081 (3)	0.5
O4C	−0.079 (2)	0.1014 (16)	0.2716 (4)	0.087 (3)	0.5
O1W	0.0000	0.5000	−0.0653 (3)	0.077 (5)	0.68 (2)

### Atomic displacement parameters (Å^2^)

**Table:**

	*U*^11^	*U*^22^	*U*^33^	*U*^12^	*U*^13^	*U*^23^
Co1	0.0367 (9)	0.0418 (9)	0.0282 (5)	−0.0009 (10)	0.000	0.000
Ca1	0.0388 (7)	0.0388 (7)	0.0274 (10)	0.000	0.000	0.000
O1	0.040 (2)	0.059 (3)	0.030 (2)	0.011 (2)	0.000 (2)	0.002 (2)
N1	0.050 (4)	0.043 (4)	0.039 (4)	0.005 (3)	−0.002 (4)	0.003 (4)
C1	0.066 (6)	0.061 (6)	0.059 (6)	−0.003 (5)	0.023 (6)	0.007 (6)
O2	0.050 (2)	0.062 (3)	0.041 (4)	0.003 (2)	−0.003 (3)	0.001 (3)
N2	0.053 (4)	0.048 (4)	0.039 (4)	0.009 (3)	0.002 (4)	0.000 (4)
C2	0.060 (6)	0.070 (7)	0.036 (5)	0.001 (6)	0.005 (5)	0.013 (5)
O3	0.049 (3)	0.065 (3)	0.054 (3)	0.018 (3)	−0.008 (3)	−0.008 (3)
C3	0.033 (5)	0.062 (6)	0.050 (6)	0.000 (4)	−0.017 (4)	−0.012 (5)
C4	0.057 (5)	0.084 (6)	0.057 (5)	−0.029 (4)	0.000 (5)	−0.001 (6)
C5	0.050 (5)	0.073 (7)	0.041 (5)	−0.013 (6)	0.012 (5)	0.006 (5)
C6	0.053 (4)	0.044 (4)	0.043 (5)	0.005 (4)	−0.006 (4)	0.004 (4)
C7	0.039 (3)	0.051 (4)	0.046 (4)	0.011 (3)	−0.009 (4)	−0.007 (4)
C8	0.034 (3)	0.043 (3)	0.037 (3)	0.001 (3)	−0.006 (4)	−0.008 (4)
C9	0.045 (4)	0.046 (4)	0.043 (4)	−0.006 (4)	0.000 (4)	−0.017 (4)
C10	0.033 (3)	0.057 (4)	0.058 (4)	0.003 (3)	0.005 (3)	−0.010 (4)
C11	0.045 (4)	0.073 (5)	0.064 (5)	0.022 (4)	0.001 (4)	−0.020 (5)
Cl1	0.072 (2)	0.072 (2)	0.0405 (11)	0.007 (3)	0.000	0.000
O1C	0.075 (7)	0.093 (7)	0.040 (3)	0.021 (6)	−0.013 (6)	−0.016 (5)
O2C	0.087 (6)	0.085 (6)	0.098 (6)	−0.009 (6)	−0.001 (6)	−0.005 (6)
O3C	0.070 (6)	0.095 (6)	0.078 (6)	−0.003 (6)	0.004 (5)	−0.006 (6)
O4C	0.100 (7)	0.092 (7)	0.071 (6)	0.017 (6)	−0.027 (6)	0.018 (5)
O1W	0.106 (8)	0.062 (7)	0.064 (7)	−0.012 (5)	0.000	0.000

### Geometric parameters (Å, º)

**Table:**

Co1—O1	2.060 (4)	C3—C5^i^	1.506 (9)
Co1—O1^i^	2.061 (4)	C3—H3A	0.9900
Co1—N1	2.192 (6)	C3—H3B	0.9900
Co1—N1^i^	2.192 (6)	C4—H4A	0.9800
Co1—N2	2.375 (6)	C4—H4B	0.9800
Co1—N2^i^	2.375 (6)	C4—H4C	0.9800
Ca1—O1^ii^	2.429 (4)	C5—C3^i^	1.506 (9)
Ca1—O1^i^	2.429 (4)	C5—H5A	0.9900
Ca1—O1^iii^	2.429 (4)	C5—H5B	0.9900
Ca1—O1	2.429 (4)	C6—C7	1.471 (9)
Ca1—O2^iii^	2.469 (4)	C6—H6A	0.9900
Ca1—O2^ii^	2.469 (4)	C6—H6B	0.9900
Ca1—O2^i^	2.469 (4)	C7—C8	1.372 (8)
Ca1—O2	2.469 (4)	C8—C9	1.467 (8)
Ca1—C9^ii^	3.182 (7)	C9—C10	1.430 (9)
Ca1—C9^iii^	3.182 (7)	C10—C11	1.320 (9)
Ca1—C9^i^	3.182 (7)	C10—H10	0.9500
Ca1—C9	3.182 (7)	C11—H11	0.9500
O1—C8	1.309 (6)	Cl1—O4C	1.345 (12)
N1—C5	1.459 (9)	Cl1—O4C^i^	1.345 (12)
N1—C1	1.486 (10)	Cl1—O1C^i^	1.413 (7)
N1—C4	1.489 (9)	Cl1—O1C	1.413 (7)
C1—C2	1.492 (10)	Cl1—O2C^i^	1.463 (14)
C1—H1A	0.9900	Cl1—O2C	1.463 (13)
C1—H1B	0.9900	Cl1—O3C	1.488 (13)
O2—C9	1.245 (7)	Cl1—O3C^i^	1.488 (13)
N2—C3	1.466 (9)	O1C—O1C^i^	0.83 (2)
N2—C6	1.472 (9)	O2C—O4C^i^	1.312 (17)
N2—C2	1.502 (9)	O2C—O3C^i^	1.651 (16)
C2—H2A	0.9900	O3C—O4C^i^	1.148 (17)
C2—H2B	0.9900	O3C—O2C^i^	1.651 (16)
O3—C11	1.334 (8)	O4C—O3C^i^	1.148 (17)
O3—C7	1.376 (7)	O4C—O2C^i^	1.312 (17)
			
O1—Co1—O1^i^	74.3 (2)	C3—N2—C6	109.3 (6)
O1—Co1—N1	121.2 (2)	C3—N2—C2	111.0 (6)
O1^i^—Co1—N1	102.9 (2)	C6—N2—C2	107.8 (7)
O1—Co1—N1^i^	102.9 (2)	C3—N2—Co1	108.1 (5)
O1^i^—Co1—N1^i^	121.2 (2)	C6—N2—Co1	117.2 (4)
N1—Co1—N1^i^	124.5 (3)	C2—N2—Co1	103.4 (5)
O1—Co1—N2	81.6 (2)	C1—C2—N2	109.3 (7)
O1^i^—Co1—N2	152.37 (19)	C1—C2—H2A	109.8
N1—Co1—N2	78.0 (3)	N2—C2—H2A	109.8
N1^i^—Co1—N2	77.0 (3)	C1—C2—H2B	109.8
O1—Co1—N2^i^	152.37 (19)	N2—C2—H2B	109.8
O1^i^—Co1—N2^i^	81.6 (2)	H2A—C2—H2B	108.3
N1—Co1—N2^i^	77.0 (3)	C11—O3—C7	119.5 (6)
N1^i^—Co1—N2^i^	78.0 (3)	N2—C3—C5^i^	111.0 (7)
N2—Co1—N2^i^	124.6 (3)	N2—C3—H3A	109.4
O1^ii^—Ca1—O1^i^	137.50 (11)	C5^i^—C3—H3A	109.4
O1^ii^—Ca1—O1^iii^	61.67 (17)	N2—C3—H3B	109.4
O1^i^—Ca1—O1^iii^	137.50 (11)	C5^i^—C3—H3B	109.4
O1^ii^—Ca1—O1	137.50 (11)	H3A—C3—H3B	108.0
O1^i^—Ca1—O1	61.67 (17)	N1—C4—H4A	109.5
O1^iii^—Ca1—O1	137.50 (11)	N1—C4—H4B	109.5
O1^ii^—Ca1—O2^iii^	123.52 (14)	H4A—C4—H4B	109.5
O1^i^—Ca1—O2^iii^	73.89 (15)	N1—C4—H4C	109.5
O1^iii^—Ca1—O2^iii^	67.05 (14)	H4A—C4—H4C	109.5
O1—Ca1—O2^iii^	96.61 (14)	H4B—C4—H4C	109.5
O1^ii^—Ca1—O2^ii^	67.05 (14)	N1—C5—C3^i^	112.4 (7)
O1^i^—Ca1—O2^ii^	96.61 (14)	N1—C5—H5A	109.1
O1^iii^—Ca1—O2^ii^	123.52 (14)	C3^i^—C5—H5A	109.1
O1—Ca1—O2^ii^	73.89 (15)	N1—C5—H5B	109.1
O2^iii^—Ca1—O2^ii^	169.1 (2)	C3^i^—C5—H5B	109.1
O1^ii^—Ca1—O2^i^	73.89 (15)	H5A—C5—H5B	107.8
O1^i^—Ca1—O2^i^	67.05 (14)	C7—C6—N2	112.8 (6)
O1^iii^—Ca1—O2^i^	96.61 (14)	C7—C6—H6A	109.0
O1—Ca1—O2^i^	123.52 (14)	N2—C6—H6A	109.0
O2^iii^—Ca1—O2^i^	90.51 (2)	C7—C6—H6B	109.0
O2^ii^—Ca1—O2^i^	90.51 (2)	N2—C6—H6B	109.0
O1^ii^—Ca1—O2	96.61 (14)	H6A—C6—H6B	107.8
O1^i^—Ca1—O2	123.52 (14)	C8—C7—O3	121.1 (6)
O1^iii^—Ca1—O2	73.89 (15)	C8—C7—C6	125.9 (6)
O1—Ca1—O2	67.06 (14)	O3—C7—C6	112.9 (6)
O2^iii^—Ca1—O2	90.51 (2)	O1—C8—C7	122.6 (6)
O2^ii^—Ca1—O2	90.51 (2)	O1—C8—C9	118.1 (6)
O2^i^—Ca1—O2	169.1 (2)	C7—C8—C9	119.0 (6)
O1^ii^—Ca1—C9^ii^	47.97 (15)	O1—C8—Ca1	44.4 (3)
O1^i^—Ca1—C9^ii^	105.63 (15)	C7—C8—Ca1	154.3 (4)
O1^iii^—Ca1—C9^ii^	108.44 (15)	C9—C8—Ca1	76.6 (4)
O1—Ca1—C9^ii^	94.80 (16)	O2—C9—C10	124.9 (7)
O2^iii^—Ca1—C9^ii^	166.55 (15)	O2—C9—C8	119.9 (6)
O2^ii^—Ca1—C9^ii^	20.98 (16)	C10—C9—C8	115.2 (6)
O2^i^—Ca1—C9^ii^	77.22 (14)	O2—C9—Ca1	45.3 (3)
O2—Ca1—C9^ii^	100.50 (14)	C10—C9—Ca1	162.3 (4)
O1^ii^—Ca1—C9^iii^	108.44 (15)	C8—C9—Ca1	76.7 (3)
O1^i^—Ca1—C9^iii^	94.80 (16)	C11—C10—C9	120.8 (7)
O1^iii^—Ca1—C9^iii^	47.97 (15)	C11—C10—H10	119.6
O1—Ca1—C9^iii^	105.63 (15)	C9—C10—H10	119.6
O2^iii^—Ca1—C9^iii^	20.98 (16)	C10—C11—O3	123.9 (7)
O2^ii^—Ca1—C9^iii^	166.55 (15)	C10—C11—H11	118.1
O2^i^—Ca1—C9^iii^	100.50 (14)	O3—C11—H11	118.1
O2—Ca1—C9^iii^	77.22 (14)	O4C—Cl1—O4C^i^	117.9 (12)
C9^ii^—Ca1—C9^iii^	156.3 (2)	O4C—Cl1—O1C^i^	128.0 (12)
O1^ii^—Ca1—C9^i^	94.80 (16)	O4C^i^—Cl1—O1C^i^	111.7 (9)
O1^i^—Ca1—C9^i^	47.97 (15)	O4C—Cl1—O1C	111.7 (9)
O1^iii^—Ca1—C9^i^	105.63 (15)	O4C^i^—Cl1—O1C	128.0 (12)
O1—Ca1—C9^i^	108.44 (15)	O1C^i^—Cl1—O1C	34.2 (8)
O2^iii^—Ca1—C9^i^	77.22 (14)	O4C—Cl1—O2C^i^	55.5 (8)
O2^ii^—Ca1—C9^i^	100.50 (14)	O4C^i^—Cl1—O2C^i^	105.5 (7)
O2^i^—Ca1—C9^i^	20.98 (16)	O1C^i^—Cl1—O2C^i^	123.9 (7)
O2—Ca1—C9^i^	166.55 (15)	O1C—Cl1—O2C^i^	89.7 (7)
C9^ii^—Ca1—C9^i^	92.42 (5)	O4C—Cl1—O2C	105.5 (7)
C9^iii^—Ca1—C9^i^	92.42 (5)	O4C^i^—Cl1—O2C	55.5 (8)
O1^ii^—Ca1—C9	105.63 (15)	O1C^i^—Cl1—O2C	89.7 (7)
O1^i^—Ca1—C9	108.44 (15)	O1C—Cl1—O2C	123.9 (7)
O1^iii^—Ca1—C9	94.80 (16)	O2C^i^—Cl1—O2C	146.3 (10)
O1—Ca1—C9	47.97 (15)	O4C—Cl1—O3C	113.5 (8)
O2^iii^—Ca1—C9	100.50 (14)	O4C^i^—Cl1—O3C	47.5 (8)
O2^ii^—Ca1—C9	77.22 (14)	O1C^i^—Cl1—O3C	110.7 (11)
O2^i^—Ca1—C9	166.55 (15)	O1C—Cl1—O3C	99.1 (10)
O2—Ca1—C9	20.99 (16)	O2C^i^—Cl1—O3C	68.0 (7)
C9^ii^—Ca1—C9	92.42 (5)	O2C—Cl1—O3C	102.7 (7)
C9^iii^—Ca1—C9	92.42 (5)	O4C—Cl1—O3C^i^	47.5 (8)
C9^i^—Ca1—C9	156.3 (2)	O4C^i^—Cl1—O3C^i^	113.5 (8)
C8—O1—Co1	134.6 (4)	O1C^i^—Cl1—O3C^i^	99.1 (10)
C8—O1—Ca1	113.4 (4)	O1C—Cl1—O3C^i^	110.7 (11)
Co1—O1—Ca1	111.99 (16)	O2C^i^—Cl1—O3C^i^	102.7 (7)
C5—N1—C1	108.2 (7)	O2C—Cl1—O3C^i^	68.0 (7)
C5—N1—C4	110.2 (7)	O3C—Cl1—O3C^i^	148.9 (11)
C1—N1—C4	109.1 (7)	O1C^i^—O1C—Cl1	72.9 (4)
C5—N1—Co1	111.2 (5)	O4C^i^—O2C—Cl1	57.7 (8)
C1—N1—Co1	111.3 (5)	O4C^i^—O2C—O3C^i^	105.8 (11)
C4—N1—Co1	106.9 (5)	Cl1—O2C—O3C^i^	56.7 (7)
N1—C1—C2	110.9 (7)	O4C^i^—O3C—Cl1	59.7 (9)
N1—C1—H1A	109.5	O4C^i^—O3C—O2C^i^	104.7 (12)
C2—C1—H1A	109.5	Cl1—O3C—O2C^i^	55.3 (7)
N1—C1—H1B	109.5	O3C^i^—O4C—O2C^i^	139.0 (15)
C2—C1—H1B	109.5	O3C^i^—O4C—Cl1	72.8 (10)
H1A—C1—H1B	108.0	O2C^i^—O4C—Cl1	66.8 (10)
C9—O2—Ca1	113.8 (4)		

Symmetry codes: (i) −*x*, −*y*, *z*; (ii) *y*, −*x*, −*z*; (iii) −*y*, *x*, −*z*.

### Hydrogen-bond geometry (Å, º)

**Table:**

*D*—H···*A*	*D*—H	H···*A*	*D*···*A*	*D*—H···*A*
C1^iv^—H1*A*^iv^···O3*C*	0.99	2.64	3.56 (2)	156
C2^iii^—H2*A*^iii^···O4*C*	0.99	2.68	3.55 (2)	147
C4^iv^—H4*C*^iv^···O1*C*	0.98	2.50	3.47 (2)	167
C5^v^—H5*A*^v^···O2*C*	0.99	2.59	3.48 (2)	148
C5^iii^—H5*B*^iii^···O4*C*	0.99	2.35	3.29 (2)	157
C6—H6*A*···O1*W*	0.99	2.35	3.23 (9)	149
C6^vi^—H6*B*^vi^···O1*C*	0.99	2.44	3.37 (2)	158
C6^vii^—H6*B*^vii^···O1*C*	0.99	2.55	3.51 (2)	164

Symmetry codes: (iii) −*y*, *x*, −*z*; (iv) −*x*+1/2, −*y*+1/2, *z*+1/2; (v) *x*−1/2, *y*−1/2, *z*+1/2; (vi) −*x*−1/2, −*y*+1/2, *z*+1/2; (vii) *x*+1/2, *y*−1/2, *z*+1/2.
